# Self-perceived knowledge, attitudes, and training needs regarding medical cannabis among health care providers and health volunteers in district health systems, Phitsanulok Province

**DOI:** 10.1186/s12875-022-01877-7

**Published:** 2022-10-21

**Authors:** Sunsanee Mekrungrongwong, Nithra Kitreerawutiwong, Orawan Keeratisiroj, Wutthichai Jariya

**Affiliations:** Faculty of Public Health, Naresuan University, 65000 Phitsanulok, Thailand

**Keywords:** Attitude, Health volunteer, Health care provider, Medical cannabis, Perceived knowledge, Training needs

## Abstract

**Background:**

Health care providers and health volunteers play an important role in the collaborative provision of patient access and care regarding medical cannabis in district health systems (DHSs) according to their roles and responsibilities. However, there is limited evidence on the self-perceived knowledge, attitudes, and training needs regarding medical cannabis use by health care providers and health volunteers in DHSs. The aim of this study was to compare self-perceived knowledge, attitudes, and training needs regarding medical cannabis among health care providers and health volunteers in Phitsanulok Province, Thailand.

**Methods:**

A cross-sectional study was conducted in Phitsanulok Province. A total of 836 participants consisting of 166 health care providers and 670 health volunteers were recruited by stratified cluster random sampling. Descriptive and inferential statistics, including independent sample t tests and chi-square tests, were employed for data analyses.

**Results:**

The results revealed that self-perceived knowledge of medical cannabis was not significantly different between the health care providers and health volunteers (p = 0.875), whereas attitudes about medical cannabis were significantly different between the two groups (p < 0.001). The mean scores of attitudes were 29.10 for the health care providers and 31.84 for the health volunteers. Regarding training needs, the health care providers proposed training topics, including adverse effects of medical cannabis (27.5%), information on prescribing cannabis drugs (20.5%) and information on prescribing drugs that contain cannabis substances (14.7%). On the other hand, health volunteers preferred to obtain training on cannabis legislation (23.5%), information on caring for patients who used drugs containing cannabis substances (21.4%), and a history of medical cannabis use (17.6%).

**Conclusion:**

In summary, to ensure effective care in the DHSs, health care providers and health volunteers should be trained to be confident in their knowledge and attitudes towards the use of medical cannabis. Training topics should be designed with consideration for the role and responsibility of each group to prepare professionals and nonprofessional to achieve patients quality and safety with respect to medical cannabis use.

## Introduction

Cannabis has been classified as a narcotic drug since 1960. It is considered an illegal item in nearly all countries [1]. However, cannabis has been legalized in many countries with many regulatory frameworks, allowing it to be used for research, medical and recreational purposes [2]. Even so, some have argued regarding the adverse effects of the substances in cannabis, which are delta-9 tetrahydrocannabinol (THC) and cannabidiol (CBD). CBD is a nonpsychoactive phytocannabinoid and is considered therapeutic for chronic illness and related symptoms [3, 4]. Although cannabis is classified as illegal and the recreational use of the drug remains illegal in some countries, according to the report of the United Nations Office on Drug and Crime (UNODC), in 2019, the prevalence of cannabis use in the world population was 3.86% [5].

In Thailand, the government started to control the use of cannabis by launching the Cannabis Act of 1934. After that, the Narcotics Act of 1979 classified cannabis as a class 5 narcotic, consistent with the convention of the United Nations, of which Thailand is a member. Since then, cannabis has been classified as a drug whose use constitutes a criminal offense as a Category V narcotic in Section 7 [6]. In 2019, the possession and use of cannabis for medical and research purposes under certain conditions was legalized for the first time in Thailand. This was to allow cannabis to be used for education, research and development for medical use. Medical cannabis could be used for patients for medical purposes by licenced health care providers, including doctors and dentists, pharmacists, traditional Thai medicine doctors, and folk healers. This practice was according to the guidelines of the Ministry of Public Health (MoPH) [7].

In July 2019, the MoPH developed the subsection of the medical cannabis service plan in the health service system to promote the accessibility of patients with serious illnesses who do not respond to conventional or Thai traditional medicines but need to receive medical care for their symptoms to medical cannabis. In addition, the recommendations on cannabis treatment and care in Thailand were published by the MoPH, covering the use for a range of conditions of both modern and traditional medicines of 16 regimens that were approved and carried out through the service plan [8]. In 2019, medical cannabis clinics were set up in 26 MoPH hospitals nationwide. The preparation of the health care workforce started from the curriculum to train health care providers in DHSs, which are networks of primary care centres and district hospitals that provide access and deliver health services to local communities. In 2020, the number of clinics that used medical cannabis was 758, including 339 conventional medicine clinics and 419 traditional Thai medicine clinics [8–10]. Then, health care providers were trained by the MoPH on medicinal cannabis and their roles.

Regarding the health care workforce in the DHSs, there are health care providers and nonprofessional health volunteers who are responsible for the health of people in the community. Health volunteers are a group of people who are trained in health care working together with health care providers at the primary care level. Health volunteers take care of people in families and the community; they are family volunteers, caregivers (CGs), and village health volunteers (VHVs) [11]. The health care provider needs to work together with the health volunteer to provide services at the patient’s home. The training for authorized medical cannabis is provided in conventional and traditional Thai medicine clinics. When medical cannabis is permitted with medical purposes at home, the patients are cared for by health volunteers; therefore, the health volunteers play an important role in observing and reporting adverse effect*s* and returning the remaining cannabidiol drug to the health care facility [10]. In addition, the MoPH provides training and communicates about medical cannabis to health volunteers only on medical conditions [9].

Evidence from the literature review illustrated that health care providers were concerned about knowledge, adverse effects, and drug abuse. They also wanted to have further training [12–16]. The pharmacists lacked knowledge, resulting in uncertain responses about adverse effects [17–19]. Other health care professionals were concerned about knowledge, drug abuse, adverse effects and drug interactions, unclear practice guidelines and the follow-up of the adverse effects of medical cannabis [20–22]. Previous studies reported that health care providers rated their knowledge of medical cannabis as poor and moderate [13, 23, 24]. Self-perceived knowledge regarding medical cannabis is one’s self-assessment of medical cannabis that helps the subjects to comprehend and form connections with new information when making decisions. Individuals who perceived their knowledge at the low level also value old and new information in a different way than thosewho perceived their knowledge at the high level [25]. Therefore, understanding self-perceived knowledge will contribute to tailor the training on medical cannabis. In addition, self-perceived knowledge, attitudes, and training needs regarding medical cannabis use by health care providers and health volunteers relevant to medical cannabis use at home in DHSs seem to be limited. This information could be beneficial in designing training to meet the needs and requirements of health care providers and health volunteers regarding medical cannabis to assure the service quality and safety of patients. This study aimed to compare self-perceived knowledge, attitudes and training needs regarding medical cannabis among health care providers and health volunteers in DHSs in Phitsanulok Province.

## Methodology

This was a cross-sectional survey study. Phitsanulok Province was purposively selected because this province has medical cannabis clinics at the tertiary care and secondary care level, which allowed us to access the sample who had experienced medical cannabis use.

### Population and sample

The population consisted of 22,422 health care providers who practice in Phitsanulok [26] and 92,823 health volunteers [27]. The sample size was calculated with the number of populations available using the formula of finite population mean by n4studies [28]. The σ is the standard deviation (S.D.); as no study on the number of health care providers in Thailand had been carried out, the S.D. was 0.5. The d was 10% of σ, with an α of 0.05, yielding 142 samples. The sample size was adjusted to minimize the nonresponse rate to 15%, resulting in 166 samples. The samples were allocated according to the population ratio in each profession according to the sampling frame. Simple random sampling was employed for the health care providers. For health volunteers, the sample size was calculated with a S.D. of 0.5, and d was 10% of σ, with an α of 0.05, resulting in 670 samples. A stratified cluster random sampling approach combined with stratified and cluster sampling methods was applied to ensure that the sample was representative of the district [29]. First, the researcher stratified the region by district. The distribution of the samples was undertaken according to the ratio of the populations in each district. Then, cluster random sampling based on subdistricts of each district was employed. Finally, the sample was subjected to simple random sampling from a sampling frame of health volunteers by random computer number generators to minimize selection bias.

For the health care providers, an anonymous questionnaire and participant information sheet were sent to them through the coordinators of the facilities who distributed the materials, reminded them, and sent all the completed questionnaires back to the researcher via postal mail. The health care providers read and understood the information of the questionnaire and participant information sheet, and then they gave consent by completing the questionnaire. Respondents were allowed two weeks to complete the questionnaire. Two reminders about *c*ompleting the questionnaire were communicated by the coordinators at the facilities one week before the due date during a two-week period. For health volunteers, the subdistrict health promotion hospital (previously called the health centre) held monthly meetings for health volunteers; therefore, the research team approached the health volunteers according to the sampling number to invite them to participate in this study. The research team made an appointment with the subdistrict health promotion hospital director to explain this project. An information sheet explaining the study was given to all participants. Consent forms were read to and discussed with all participants by the research team to obtain voluntary informed consent. Health volunteers who met the inclusion criteria and were willing to participate were invited to complete the questionnaire in the meeting rooms of the facilities, and permission was obtained from the directors of the facilities. The research team stood by outside the room to ensure a neutral atmosphere for the respondents. If the respondents asked for assistance, the researchers facilitated their requests. The inclusion criteria health care providers or health volunteers in the community responsible for the health of the population in the DHS. The exclusion criterion was refusal to give informed consent. All methods were performed in accordance with the relevant guidelines and regulations.

### Instruments

The research tool of this study was a questionnaire with four parts. The details of each part are as follows.

Part 1: Demographic characteristics. For the health care providers, there were eight items, including sex, age, profession, working duration in health care, work department, training, experience and knowledge of medical cannabis usage. For the caregivers, the number of items was also eight, including sex, age, career, educational level, experience and knowledge of medical cannabis usage, and interest in learning about medical cannabis.

Part 2: Self-perceived knowledge, which was developed based on the reviewed literature [15, 17]. This part consisted of 8 items related to the appropriate amount of use, actual amount of use, methods of use, patterns of use, legislation, adverse effects and use of medical cannabis at home. It aimed to evaluate self-perceived knowledge regarding the use of medical cannabis. A five-point Likert scale (5 referred to an excellent level of self-perceived knowledge, whereas 1 referred to a poor level of self-perceived knowledge) was applied.

Part 3: Attitudes towards using medical cannabis with 10 items developed based on the previous literature [15, 17]. These items were assessed via a five-point Likert scale (5 referred to strongly agree, whereas 1 referred to strongly disagree).

Part 4: Training needs were assessed by four multiple choice and short answer items. The items consisted of the readiness to give advice on medical cannabis usage, training needs regarding medical cannabis use, training duration, and suggestions for the development of medical cannabis use.

The content validity was examined by five experts, and the value of the index of item objective congruence was 0.80-1.00. The reliability of the questionnaire was assessed by pilot testing with 30 health care providers and 30 health volunteers in Sukhothai Province. The Cronbach’s alpha coefficients for perceived knowledge and attitudes among health care providers were 0.91 and 0.85, respectively, and those for the health volunteers were 0.78 and 0.80, respectively. This research was approved by the Naresuan University Institutional Review Board (Project number: P10087/63). The participants consented to voluntary participation and were not compensated.

### Data analysis

Descriptive statistics were used to present demographic characteristics, self-perceived knowledge, attitudes and training needs for medical cannabis use. Inferential statistics of t tests for independent samples were used to compare the mean scores of self-perceived knowledge and attitudes towards medical cannabis use between health care providers and health volunteers. In addition, the chi square test was used to compare the responses of ordinal variables (items on perceived knowledge and attitudes) and the nominal variables of health care providers and health volunteers. The significance level was set at α = 0.05. The training needs regarding the use of medical cannabis were grouped into themes and presented as percentages for each theme.

## Results

### Descriptive characteristics of the sample

There were 836 participants in this study, including 166 health care providers (19.9%): 17.10% were health care providers in conventional medicine, 2.8% were health care providers in traditional Thai medicine, and 670 were health volunteers (80.1%). Most respondents were female (77.5%). The average age was 48.09 years (S.D.=14.07). Most of them had no experience using cannabis for medical purposes (96.5%).

Among 166 health care providers, the majority were pharmacists (53 subjects, 31.9%). Their average age was 35.57 years (S.D.=7.97), and most had worked in the health profession for 11.17 years (S.D.=7.68). Most health care providers of conventional and traditional Thai medicine had not received training in using medical cannabis (66.9%), whereas the number of those who received the training was smaller (33.1%). The majority of them had no experience using medical cannabis (91.0%), and most of them had access to information about medical cannabis use from the internet (27.5%).

Most of the health volunteers were agriculturalists (57.9%) and had completed primary school (85.0%), with a mean age of 51.19 years (S.D.=13.52). Most had no experience in using medical cannabis (97.9%) and had learned to use medical cannabis from television and radio (42.46%). The percentage of health volunteers who were interested in learning to use medical cannabis was 86.4%.

### Comparison of the overall scale of self-perceived knowledge and attitudes towards medical cannabis use

Table 1 presents the comparison of self-perceived knowledge and attitudes towards using medical cannabis among health care providers and health volunteers at a significance level of 0.05. The results revealed no significant difference in self-perceived knowledge between the two groups (p = 0.875). For attitudes, there were significant differences (p < 0.001). Table 1.

**Table 1:** Self-perceived knowledge, attitudes, and training needs regarding medical cannabis among health care providers and health volunteers

Variables	Mean (S.D.)	Mean diff. (95% CI)	p-value^*^
Self-perceived knowledge regarding the use of medical cannabis			
Health care providers	17.16 (7.75)	0.103 (-1.055 to 1.261)	0.875
Health volunteers	17.06 (6.55)		
Attitude toward the use of medical cannabis			
Health care providers	29.10 (4.52)	2.739 (2.024 to 3.455)	< 0.001*
Health volunteers	31.84 (4.12)		

* p-value < 0.05

### Comparison of each item of self-perceived knowledge about medical cannabis

Regarding the items of self-perceived knowledge about medical cannabis use, the health care providers and health volunteers were significantly different in the appropriate amount of medical cannabis (p = 0.031), method of medical cannabis use (p < 0.001), legislation of medical cannabis use (p = 0.004), adverse effects, warning signs and caution for patients in medical cannabis use (p < 0.001), and reports of complications about medical cannabis use (p = 0.002). Most health care providers responded that they had low to very low self-perceived knowledge about medical cannabis use (60–70%). However, for Item 6, adverse effects and warning signs and caution for patients in medical cannabis use, most had moderate self-perceived knowledge about medical cannabis use. Regarding the health volunteers, 50–60% had low to very low self-perceived knowledge about medical cannabis use (Table 2). Table 2

**Table 2:** The comparison of the items of self-perceived knowledge regarding the use of medical cannabis

Self-perceived knowledge regarding the use of medical cannabis	Number (%)					p-value
	**Very low**	**Low**	**Medium**	**High**	**Very high**	
**Item 1** Appropriate amount of medical cannabis						0.031*
Health care providers	63 (38.0)	49 (29.5)	36 (21.7)	16 (9.6)	2 (1.2)	
Health volunteers	177 (26.4)	242 (36.1)	190 (28.4)	52 (7.8)	9 (1.3)	
**Item 2** Amount of using medical cannabis						0.126
Health care providers	66 (39.8)	48 (28.9)	37 (22.3)	13 (7.8)	2 (1.2)	
Health volunteers	224 (33.4)	267 (39.9)	135 (20.1)	38 (5.7)	6 (0.9)	
**Item 3** Methods and administration of using medical cannabis						< 0.001*
Health care providers	53 (31.9)	51 (30.7)	38 (22.9)	22 (13.3)	2 (1.2)	
Health volunteers	173 (25.8)	261 (39.0)	137 (20.4)	43 (6.4)	56 (8.4)	
**Item 4** Similarity and difference in administration of using medical cannabis through various methods						0.088
Health care providers	66 (39.8)	45 (27.1)	42 (25.3)	12 (7.2)	1 (0.6)	
Health volunteers	221 (33.0)	226 (33.7)	136 (20.3)	80 (11.9)	7 (1.0)	
**Item 5** Legislation in using medical cannabis						0.004*
Health care providers	59 (35.5)	51 (30.7)	34 (20.5)	19 (11.4)	3 (1.8)	
Health volunteers	196 (29.3)	226 (33.7)	159 (23.7)	39 (5.8)	50 (7.5)	
**Item 6** Adverse effects, warning signs and caution for patients to use medical cannabis						< 0.001*
Health care providers	37 (22.3)	43 (25.9)	53 (31.9)	28 (16.9)	5 (3.0)	
Health volunteers	217 (32.4)	226 (33.7)	147 (21.9)	47 (7.0)	33 (4.9)	
**Item 7** Caring for patients being treated with medical cannabis						0.283
Health care providers	63 (38.0)	54 (32.5)	33 (19.9)	14 (8.4)	2 (1.2)	
Health volunteers	236 (35.2)	276 (41.2)	112 (16.7)	39 (5.8)	7 (1.0)	
**Item 8** Reporting for complication and risks when using medical cannabis						0.002*
Health care providers	58 (34.9)	47 (28.3)	39 (23.5)	19 (11.4)	3 (1.8)	
Health volunteers	245 (36.6)	270 (40.3)	114 (17.0)	34 (5.1)	7 (1.0)	

* p-value < 0.05

### Comparison of each item of attitudes towards medical cannabis use

When comparing the responses of each item regarding attitudes towards medical cannabis use, health care providers and health volunteers showed significant differences for all items (p < 0.05). Among the health care providers and health volunteers whose answers were “Agree” and “Strongly agree”, they were significantly different in the following items: 1) All patients could be treated with medical cannabis (2.4% vs. 18.8%); 2) Medical cannabis should not be the first drug of choice for treatment (80.1% vs. 27.9%); 3) The practice guideline of the Ministry of Public Health is clear (18.1% vs. 29.7%); 4) For appropriate treatment of the patient, the primary caregiver of the patient who provides medical cannabis should receive training (90.3% vs. 57.7%); 5) When under prescription or with suggestion from health care providers who passed the training, medical cannabis is safe (65.7% vs. 50.7%); 6) Using medical cannabis may lead to addiction to other drugs (22.3% vs. 17.6%); 7) Using medical cannabis might cause crime (16.8% vs. 13.9%); 8) I am concerned about the use of medical cannabis at the home of the patients (54.8% vs. 31.1%); 9) Medical cannabis may relieve symptoms of chronic disease (10.2% vs. 23.8%); and 10) Patients who use medical cannabis are at risk of having hallucinations (21.6% vs. 13.2%) (Table 3).

**Table 3:** The comparison of the items of attitudes toward the use of medical cannabis

Attitude	Number (%)										p-value		
	**Strongly disagree**		**Disagree**		**Unsure**		**Agree**		**Strongly agree**				
**Item 1 A**ll patients could be treated with medical cannabis.											< 0.001*		
Health care providers		65 (39.2)		74 (44.6)		23 (13.9)		4 (2.4)		0			
Health volunteers	104 (15.5)		179 (26.7)		261 (39.0)		111 (16.6)		15 (2.2)				
**Item 2** Medical cannabis should not be the first drug of choice for treatment.											< 0.001*		
Health care providers	3 (1.8)		5 (3.0)		25 (15.1)		54 (32.5)		79 (47.6)				
Health volunteers	85 (12.7)		156 (23.3)		242 (36.1)		108 (16.1)		79 (11.8)				
**Item 3** The practice guideline of Ministry of Public Health is clear.											0.007*		
Health care providers	16 (9.6)		37 (22.3)		83 (50.0)		29 (17.5)		1 (0.6)				
Health volunteers	65 (9.7)		149 (22.2)		257 (38.4)		163 (24.3)		36 (5.4)				
**Item 4** For appropriate treatment of the patient, the primary care giver of the patient with medical cannabis should have received the training.											< 0.001*		
Health care providers	0		1 (0.6)		15 (9.0)		58 (34.9)		92 (55.4)				
Health volunteers	52 (7.8)		81 (12.1)		151 (22.5)		192 (28.7)		194 (29.0)				
**Item 5** When under prescription or with suggestion from healthcare providers who passed the training, the medical cannabis is safe.											< 0.001*		
Health care providers	0		7 (4.2)		50 (30.1)		73 (44.0)		36 (21.7)				
Health volunteers	53 (7.9)		86 (12.8)		191 (28.5)		212 (31.6)		128 (19.1)				
**Item 6** Using medical cannabis may lead to addicting to other drugs.											< 0.001*		
Healthcare providers	6 (3.6)		57 (34.3)		66 (39.8)		25 (15.1)		12 (7.2)				
Health volunteer	99 (14.8)		194 (29.0)		259 (38.7)		100 (14.9)		18 (2.7)				
**Item 7** Using medical cannabis might cause crime.											0.034*		
Health care providers	21 (12.7)		78 (47.0)		39 (23.5)		19 (11.4)		9 (5.4)				
Health volunteers	109 (16.3)		246 (36.7)		222 (33.1)		71 (10.6)		22 (3.3)				
**Item 8** you are concerned with the use of medical cannabis at home of the patients.											< 0.001*		
Health care providers	1 (0.6)		15 (9.0)		59 (35.5)		54 (32.5)		37 (22.3)				
Health volunteers	66 (9.9)		141 (21.0)		255 (38.1)		152 (22.7)		56 (8.4)				
**Item 9** Medical cannabis may relieve symptoms of chronic disease.											0.002*		
Health care providers	27 (16.3)		50 (30.1)		72 (43.4)		12 (7.2)		5 (3.0)				
Health volunteers	84 (12.5)		155 (23.1)		271 (40.4)		137 (20.4)		23 (3.4)				
**Item 10** Patients with medical cannabis are in risk of having hallucination.											< 0.001*		
Health care providers	6 (3.6)		42 (25.3)		82 (49.4)		19 (11.4)		17 (10.2)				
Health volunteers	108 (16.1)		213 (31.8)		261 (39.0)		66 (9.9)		22 (3.3)				

* p-value < 0.05

### Training needs regarding medical cannabis

Regarding the training needs for medical cannabis use, most health care providers wanted to receive training regarding the adverse effects of using medical cannabis, prescription of medical cannabis, prescription of medicine with cannabis substances, legislation regarding cannabis and cannabis oil extract (27.5%, 20.5%, 14.7%, 12.4% and 12.0%, respectively). Most of the health volunteers wanted to receive training on the legislation regarding medical cannabis use, prescription of medicine with cannabis substances, history of treatment with medical cannabis, adverse effects of treatment with medical cannabis and compounding medicine with cannabis (23.5%, 21.4%, 17.6%, 14.8% and 10.3%, respectively). Only 3.5% of the health volunteers were interested in receiving training on using cannabis oil extract.

## Discussion

Overall, the self-perceived knowledge of medical cannabis use between the health care providers and health volunteers was not significantly different (p = 0.875). From the perspective of the health care providers, medical cannabis use for each individual was different due to the difference in biological factors, diagnosis, and condition. For example, providers wanted to have knowledge about the appropriate use of cannabis for medical treatment, consideration of whether current treatment with medical cannabis may relieve symptoms of chronic disease or chronic conditions, confidence in using good-quality herbal products, knowledge of the contraindications of using medical cannabis, medically certifies evidence that products are of good quality and safe, and clear indications and precautions regarding medical cannabis use [30]. A previous study on the knowledge regarding medical cannabis among students used informal sources such as blogs, websites, and the internet [31]. This result is in line with the implementation of the MoPH provides training and communicates about medical cannabis to health volunteers [9]. From this, it is not surprising that the overall self-perceived knowledge of medical cannabis use of the health care providers was not different from that of the health volunteers.

The self-perceived knowledge of the health care providers and the health volunteers was low (62.7%, mean 17.16, S.D.=7.75 for the health care providers; 63.0%, mean 17.06, S.D.=6.55 for the health volunteers). This is in agreement with the results of a previous study showing that medical personnel were worried about issues such as knowledge [12, 13], drug abuse, and adverse drug events [14, 16]. The pharmacists reported a low level of self-perceived knowledge [17–19]. They perceived that they lacked information on pharmacology, pharmacokinetics and pharmacodynamics in medical cannabis use [18]. In addition, pharmacists accepted a lack of knowledge regarding the legislation and process of prescribing, planning and distributing cannabis [18, 19]. From a survey of pharmacists in Minnesota, United States, pharmaceutical students would like to have medical cannabis in their curriculum [17]. Most pharmacists learned about medical cannabis themselves online [19]. Most nurses and other health professionals reported having poor self-perceived knowledge about endocannabinoids and the pharmacology of endocannabinoids [21, 22]. According to them, general knowledge on the treatment process with medical cannabis, such as using cannabis, differences in the formulation, legislation, accessibility and distribution was also poor [20–22]. The main obstacle to the provision of medical cannabis advice to patients is the lack of standards of practice [20]. This group of providers desired to increase their knowledge by formal training or continuing education [21, 22]. However, the results of this study are a self-assessment of the use of medical cannabis, which is considered the starting point to fill the gap in the design of training content for health care providers and health volunteers.

With consideration of the items of self-perceived knowledge, the responses of health care providers were significantly different from those of health volunteers in terms of the appropriate dosage of cannabis, methods of using cannabis, relevant legislation, adverse effects, warning signs and precautions, and reports on complications and risks when using medical cannabis. This is consistent with the recommendations of the World Health Organization [30] and the study by Prommarat [32] that stated that we should consider comorbidities and side effects when prescribing medical cannabis to palliative care patients. They should be informed of the quality of life after using medical cannabis extract. The results of this study are also in agreement with the finding that most health care providers were not sure whether the administration of medical cannabis follows government policy. Half of the respondents would not like to answer questions asked by the patients regarding medical cannabis use, but they still would like to review the evidence supporting medical cannabis use [15]. The findings are also consistent with the results in this study that only one-third of the respondents passed the training on medical cannabis use (33.1%), and most had no experience in using medical cannabis, including prescribing, dispensing, and compounding medicine when taking care of patients (91.0%).

The majority of the respondents did not have experience using medical cannabis (97.9%). Both health care providers and health volunteers revealed the need to be trained on using medical cannabis in various aspects, including the adverse effects of using medical cannabis, prescribing medicine with cannabis or cannabis components, legislation regarding cannabis, using cannabis oil extract, history of using cannabis in medical treatment and compounding medicine with cannabis. This is consistent with the study finding that patients with Alzheimer’s disease believed that health care providers should propose using cannabis oil in treating them. Most of them searched for information regarding cannabis oil online, and only 67.0% consulted doctors regarding the use of cannabis oil [33]. Therefore, the government should provide knowledge to health care providers. This could result in the appropriate use of medical cannabis. The content of knowledge relevant to distributing medicine, controlling the advertisement, monitoring illegal trading and transferring knowledge to the health care providers of both conventional and traditional medicine would be beneficial. In addition, guidelines for diagnosing and prescribing medical cannabis, monitoring medical cannabis use, preventing drug abuse, and providing an appropriate patentability system need to be provided.

Regarding the attitudes towards medical cannabis use among the health care providers and health volunteers, their overall attitudes were significantly different (p < 0.05). The health volunteers had more positive attitudes than the health care providers. The reason why the health volunteers were familiar with cannabis use might be because cannabis has been used in the community since their ancestors in Thai traditional medicine [31]. Moreover, after one year of legalization, Chaophraya Abhaibhubejhr Hospital, a public hospital that is a centre of integration between Western medicine and traditional Thai medicine, formulated 16 formulae with a second category of medical cannabis for insomnia, stroke, muscle spasm, poor appetite, and chronic pain [34].

When considering the attitude items, the attitudes of the health care providers whose answers were “Strongly agree” and “Agree” were stronger than those of the health volunteers. These attitude items were as follows: (1) medical cannabis should not be used as the first drug of choice; (2) primary caregivers of patients who use medical cannabis should receive training for appropriately taking care of the patients; and (3) using medical cannabis is safe when it is prescribed or suggested by trained health care professionals. This is consistent with the WHO [30] in that heath care providers are likely to consider empirical evidence in using medical cannabis as it might cause addiction to other drugs, crimes, and risks of hallucinations. They were also concerned about the use of medical cannabis by the patients at home. This result is in agreement with the findings of the health care providers who were concerned about the risks of using cannabis in an uncontrollable situation [14, 16]. From these findings, guidelines for medical cannabis use at home are essential.

The strength of this study is that it was conducted in the province that has implemented medical cannabis in its DHSs, and this situation led to the experience of the sample. The selection bias of the sample was minimized by probability sampling. However, this study had the following limitations: the sample was limited to one province that had a medical cannabis clinic in a DHS; therefore, generalizability must be performed with caution in a similar setting. In addition, assessing perceived knowledge and attitudes using an opinionnaire may inaccurately denote a true representation of knowledge but rather a perception of knowledge that is perhaps influenced by external factors. However, the information of self-perceived knowledge about medicinal cannabis of health care providers and health volunteers highlighted the need for further training and education to access information. Notwithstanding the uniqueness of the setting, the results can contribute to the design of training courses for health care providers and health volunteers to meet their needs for collaborative work in DHSs and ensure the safety of medical cannabis use.

## Conclusion

To increase confidence in their knowledge and positive attitudes, heath care providers and health volunteers should receive different training on medical cannabis, depending on their contexts and roles. Health care providers might be specifically trained on the clinical use of medical cannabis, such as dosage, assessment of patients and indications for cannabis use. Health volunteers should be trained on the use, methods, observation of symptoms and adverse effects and report on complications and risks when using cannabis in patients’ homes. This should lead to the development of a medical cannabis training manual for health care providers and health volunteers. In addition, an assessment of the knowledge of medical cannabis use of heath care professionals who are involved in cannabis usage should be conducted in further studies.

## Data Availability

The datasets of this study are available from the corresponding author upon reasonable request due to the protection under the terms of the Naresuan University Ethical Committee for dissemination.

## Abbreviations

CBD: Cannabidiol.
CG: Caregiver.
DHS: District health system.
MoPH: The Ministry of Public Health.
S.D.: Standard deviation.
THC: Delta-9 tetrahydrocannabinol.
UNODC: The United Nations Office on Drug and Crime.
VHV: Village health volunteer.
